# Laser Desorption of Explosives from the Surface of Different Real-World Materials Studied Using C_2_Cl_6_-Dopant-Assisted Ion Mobility Spectrometry

**DOI:** 10.3390/molecules29184482

**Published:** 2024-09-21

**Authors:** Emanuel Maťaš, Matej Petrík, Martin Sabo, Štefan Matejčík

**Affiliations:** 1 Faculty of Mathematics, Physics and Informatics, Comenius University in Bratislava, 842 48 Bratislava, Slovakia; emanuel.matas@fmph.uniba.sk; 2 Faculty of Informatics and Information Technologies, Slovak University of Technology in Bratislava, 842 16 Bratislava, Slovakiamartin.sabo@masatech.sk (M.S.); 3 MaSa Tech s.r.o., Sadová 3018/10, 916 01 Stará Turá, Slovakia

**Keywords:** ion mobility spectroscopy, explosive detection, laser desorption, trace analysis, security checkpoint

## Abstract

A highly efficient and sensitive ion mobility spectrometry (IMS) system with laser desorption sampling was applied for rapid explosive detection using different surface materials. This portable IMS detector, powered by a battery, offers mobility and is suitable for use in the field or combat zones. The laser desorption (LD) sampling of common explosives (Trinitrotoluene—TNT; Dinitrotoluenes—DNTs; Hexogene—RDX; pentaerythritol tetranitrate—PETN; plastic explosives—Compound 4 (C-4) and Semtex) on a wide range of common surface materials, such as metal, ceramic, plastic, glass, drywall, paper, wood, and textiles, was studied. Successful detection was achieved on nearly all surfaces except flammable materials (paper, wood, and textiles). The limit of detection (LOD) was determined for each explosive and specific surface, demonstrating an impressive LOD of 7 ng/mm^2^ for TNT. RDX, C-4, PETN, and Semtex achieved LOD values of 15 ng/mm^2^, while DNTs showed an LOD of approximately 50 ng/mm^2^.

## 1. Introduction

The situation in Ukraine and the Middle East is extremely complex, unstable, and dangerous. The increasing number of terrorist attacks in the world’s capitals is also causing fear and concern. Therefore, the protection of citizens must be the top priority in global national security. The identification and detection of trace amounts of explosives or chemical warfare agents are crucial in various security areas such as airports, borders, and railway stations, as well as for forensic purposes at crime sites. The detection of explosives is crucial not only in war zones or hazardous areas to prevent potential threats and enhance the safety of soldiers [1] but also to ensure the well-being of citizens residing in post-war zones [2]. Various methods have been employed to detect explosives, such as animal olfactory systems [3], the colorimetric technique [4,5,6], immunosensors [7], nanotechnology sensors [8], spectroscopy methods [9,10,11,12,13], mass spectrometry (MS) [14,15], and ion mobility spectrometry (IMS) [14,15,16,17,18,19,20,21,22]. Among these techniques, IMS stands out as a reliable analytical method for detecting explosives, chemical warfare agents [14,19,23,24,25], and illegal drugs [23,26,27].

Ion mobility spectrometry is a fast ion separation technique that is based on the interaction of ions with the neutral molecules of the drift gas in a weak electric field [28,29]. The advantages of IMS are its fast response (ms) [28], high sensitivity (parts per billion/quadrillion (ppb; ppq) level) [22], ability to operate at atmospheric pressure, simple and cheap design, and good portability [28,29]. Therefore, IMS has been a key instrument in detecting explosives, drugs, and chemical warfare agents at airports for many years [1,25]. The IMS instrument consists of several parts, including the drift region, ionization source, reaction region, and detector [28,29]. The heart of IMS is the drift tube, which consists of the reaction and the drift regions. The homogeneous electric field is created by a series of conductive rings isolated by Teflon rings [28,29]. Vapors of samples are introduced into the reaction region, where the sample molecules are ionized via chemical ionization. The new ions formed in the reaction region are injected into the drift region for separation by a shutter grid. The Faraday plate (detector) is placed at the end of the drift tube and shielded by the aperture grid. The separation of ions in the drift tube depends on the mass, shape, size, and charge of the ions [28]. The IMS can operate in both polarities, positive and negative.

The most common ionization source used in IMS is the radioactive one, based on the isotope ^63^Ni [23,24,30,31]. Other ionization methods such as electrospray ionization (ESI) [18], photoionization source [32], matrix-assisted laser desorption ionization (MALDI) [33], direct analysis in real time (DART) [34,35], and corona discharge (CD) [15,36,37,38,39,40,41] are also commonly used.

The present instrument was equipped with a corona discharge (CD) ionization source. CD is a non-radioactive ionization source that can generate positive and negative ions. However, in negative-polarity CD, very stable negative reactant ions (RI) can be formed in ambient air, such as N_2_O_2_^−^, NO_3_^−^, CO_3_^−^, and HNO_3_^−^ [37,39,42,43], with high electron affinity (EA). These ions can only ionize, by charge transfer, samples with a higher EA than RI. Therefore, these ions cannot effectively ionize explosives. Ross and Bell presented a remarkably simple and effective method by designing the reverse-gas-mode CD [38]. In their design, the carrier gas flows through the CD gap, removing the neutral particles generated in the CD and causing the RI to change to O_2_^−^CO_2_∙(H_2_O)_n_ (n = 1, 2, 3, …) [37,39,43], with a significantly lower EA [38].

Asbury et al. [44] studied explosives using the electrospray–IMS technique. They measured the following typical values of reduced ion mobility (K_0_): 1.48 for TNT, 1.40 for RDX, and 1.62 cm^2^·V^−1^·s^−1^ for 2,6-DNT. The corresponding LODs for these substances were 26, 40, and 15 ppb, respectively [44]. Burykov et al. [45] reported a very interesting detection limit in a direct analysis of the vapors of TNT, PETN, and 2,4-DNT at around 0.015 pg/mL. Babis et al. [46] reported a picogram LOD for all investigated explosives using direct vapor sample analysis via miniature IMS. The average vapor concentrations at the LOD were 0.71 ppb for TNT, 80 ppb for RDX, 180 ppb for PETN, 738 ppb for 2,4-DNT, and 170 ppb for 2,6-DNT [46]. 

An effective sampling technique is important for the detection of low-volatility compounds (explosives). Thermal or laser desorption (TD/LD) is a frequently used technique for IMS analyses using various materials [14,15,19,20,22,47]. In the TD technique, the surface of the investigated sample is heated (typically 60–280 °C) to evaporate molecules that are subsequently ionized in the reactant region [14,19,47].

Popov et al. [47] investigated the direct detection of explosives such as TNT, RDX, and PETN using the TD-IMS technique on cotton swabs and in particulates. The TD temperatures for TNT, RDX, and PETN were set to 100, 150, and 150 °C, respectively. LODs of 10 ng (TNT), 30 ng (RDX), and 10 ng (PETN) were achieved, regardless of the surface material [47]. Najarro et al. [19] detected the residues collected from explosives on a sampling swipe using TD from a swab. They obtained optimal temperatures for the highest IMS sensitivity; these were 80 °C for TNT, 100 °C for PETN, and 160 °C for RDX. For plastic-bonded explosives, the temperatures were 100 °C for PETN in Semtex, 160 °C for RDX in C-4, and 160 °C for RDX in Semtex [19]. Sabo et al. [14] desorbed TNT using a heated stream of N_2_ (140 °C) from the tip of a stainless-steel needle. The LOD was 350 pg [14]. Chouyyok et al. [48] explored the fundamental attributes of muslin and fiberglass cloth for surface sampling in explosive detection. The desorption of explosives was conducted using TD. They detected explosives such as TNT, RDX, PETN, and other materials with a very good signal response. Kosterev et al. [22] demonstrated a portable IMS with dopant-assisted laser ionization (YAG:Nd^3+^ laser). The explosives were evaporated from the sample in a thermostatic shell with a heater. The best calculated LODs they obtained for RDX and PETN, with toluene dopant, were 50 and 760 ppq, respectively [22]. Li et al. [27] utilized the TD technique combined with a miniature DT-IMS instrument to detect explosives on a Nomex swab. The temperature of the thermal desorption sampler was set to 200 °C. TNT, RDX, and PETN were clearly detected and identified. The LOD for TNT was less than 0.1 ng [27].

In another study, Sabo et al. [15] demonstrated the application of a laser diode desorption technique connected with an IMS instrument to perform a surface analysis of explosives. Utilizing this method, they achieved very high LODs for TNT, RDX, and PETN from a stainless-steel needle, specifically 0.6 pg, 2.8 pg, and 8.4 pg, respectively [15]. Ehlert et al. [20] used a pulsed Nd:YAG laser to facilitate the surface desorption of several investigated explosives. They obtained good LODs for all explosives detected on the aluminum foil: TNT (1 ng), RDX (25 ng), and PETN (10 ng) [20].

In this manuscript, we present a study of LD combined with an IMS negative CD ionization source doped with an admixture of C_2_Cl_6_. Introducing the dopant to the ionization source changes the formation of reaction ions to Cl^−^, significantly enhancing the sensitivity of the analytical device to explosive compounds [15,25,49]. This modification improves ionization efficiency, resulting in improved detection limits for target analytes. Direct laser desorption and detection of TNT, RDX, PETN, C-4, Semtex, and 2,4-DNT, 3,4-DNT, and 2,6-DNT are studied on several real-world materials (aluminum, stainless steel, ceramic, PVC, glass, drywall, paper, wood, cotton, and denim) using a diode laser with a wavelength of 532 nm. A portable IMS device is used in this work, powered by a battery, suitable for use in many fields. This portable IMS device can be used to detect explosives in war zones or trace amounts of chemicals used in the preparation of explosives in illegal laboratories.

## 2. Results and Discussion

### 2.1. Detection of Explosives

**Figure 1:**
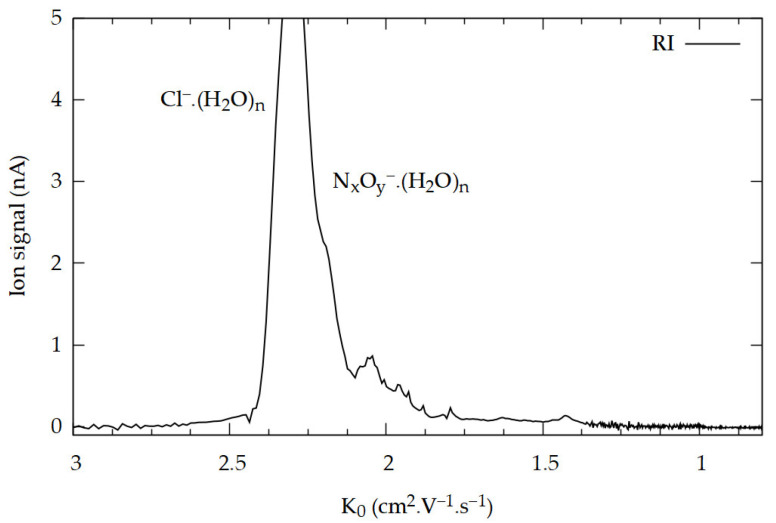
The IMS spectrum of negative CD with dopant C_2_Cl_6_ contains RIP Cl^−^∙(H_2_O)_n_ (dominant peak with K_0_ = 2.30 cm^2^·V^−1^·s^−1^) and small admixture of NO_x_^−^ (peaks on the right side).

An IMS instrument equipped with a CD ionization source was operated in the reverse gas flow mode at 60 °C. Purified air was used as the drift gas, and the addition of a small admixture of dopant gas C_2_Cl_6_ resulted in the formation of a dominant reactant ion (RI) peak for Cl^−^∙(H_2_O)_n_ (n = 0, 1, 2, 3, …) with a reduced ion mobility value (K_0_) of 2.30 cm^2^·V^−1^·s^−1^, as well as an auxiliary peak for N_x_O_y_^−^∙(H_2_O)_n_ (the bulk on the right side) [14,39,43], as shown in Figure 1. The negative ions and their water clusters appear in the IMS spectrum as a single peak (RI) due to the equilibrium between cluster formation and the dissociation of clusters in the IMS drift tube [43]. The purpose of the dopant gas is to enhance the sensitivity and selectivity of the instrument for explosive detection [25,50].

The explosive samples were prepared on the surfaces of interest and desorbed using a focused diode laser beam. The desorbed analytes were sucked into the IMS reaction region for ionization via APCI by Cl^−^∙(H_2_O)_n_ reactant ions [51]. In the case of nitrotoluene samples’ TNT and DNT isomers, the ionization proceeds via a deprotonation reaction (1). The [TNT−H] ^−^ and [DNT−H] ^−^ ions are formed via a proton abstraction reaction between a nitrotoluene compound and chloride reactant ions and are characterized by excellent chemical and thermal stability [25,51]. In the case of nitroamine explosives such as RDX, C-4, PETN, and Semtex, the major product ions are adduct ions [RDX + Cl] ^−^ for RDX and C-4, and [PETN + Cl] ^−^ for PETN and Semtex. These product ions are formed via a three-body associative ion–molecule attachment reaction between Cl^−^ ions and nitroamine compounds, as shown in reaction (2), where N_2_ serves as the third body [25,51].

Cl^−^ + M → Cl^−^ ∙ M → (M − H) ^−^ + HCl, (1)

Cl^−^ + M + N_2_ → Cl^−^ ∙ M + N_2_. (2)

**Figure 2:**
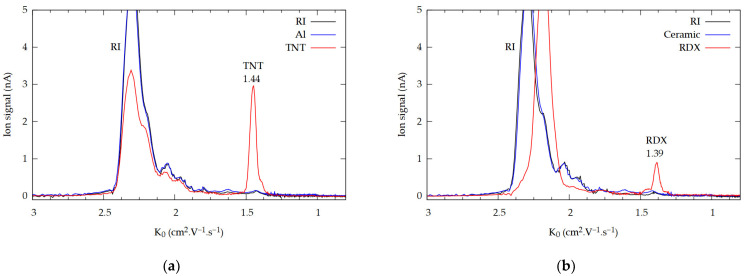

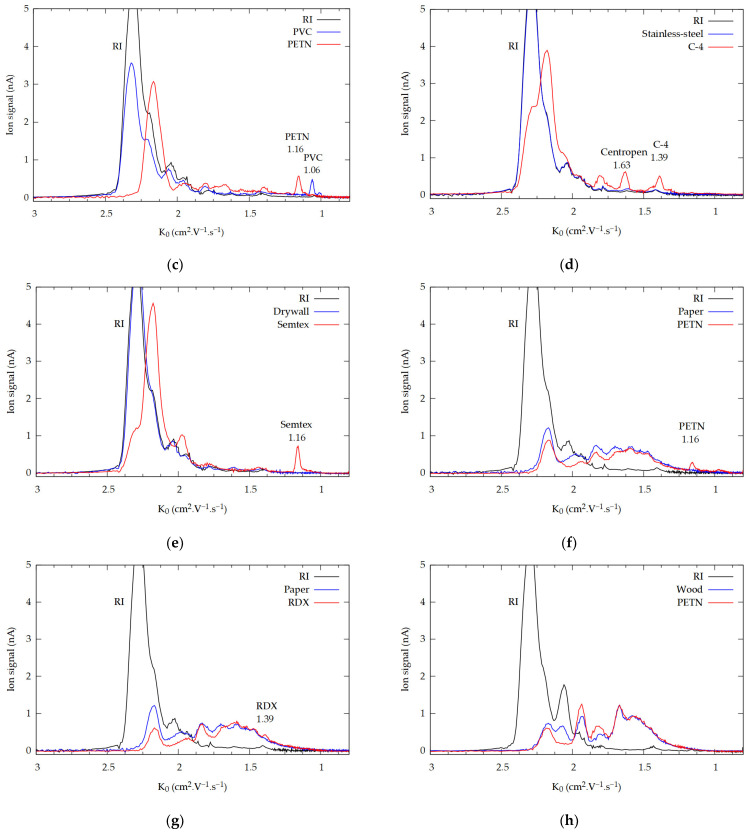
Sample of selected IMS spectra of explosives from different surface materials. (**a**) TNT from aluminum; (**b**) RDX from ceramic; (**c**) PETN from PVC; (**d**) C-4 from stainless steel; (**e**) Semtex from drywall; (**f**) PETN from paper; (**g**) RDX from paper; and (**h**) PETN from wood. The surface concentrations were not calculated.

Sample IMS spectra of the individual explosives from a specific material are shown in Figure 2. The black curve represents the spectra of RI, the blue curve represents the IMS response to the specific material after laser irradiation, and the red curve represents the IMS spectrum of the explosive desorbed from the investigated material. 

The IMS response to the TNT, RDX, and PETN resulted in the formation of peaks with reduced ion mobilities of 1.44, 1.39, and 1.16 cm^2^·V^−^·s^−1^, respectively (Figure 2a–c, f). In the case of C-4, the IMS response was identical to that of RDX (1.39 cm^2^·V^−1^·s^−1^), and in the case of the Semtex, it was similar to that of PETN (1.16 cm^2^·V^−1^·s^−1^), as expected. The present values of reduced ion mobilities are comparable to those reported in earlier studies [25,44,51]. Figure 2 only shows sample results for a comprehensive set of IMS spectra corresponding to each explosive detected from each surface material; please refer to the Supplementary Materials for more spectra.

In addition to TNT, we carried out studies of isomers of DNTs. Characteristic peaks were detected for each isomer (1.55 for 2,4-DNT, 1.52 for 3,4-DNT, and 1.46 cm^2^·V^−1^·s^−1^ for 2,6-DNT; please see Figures S6–S8 in each section of the Supplementary Materials). In the case of 3,4-DNT, three specific peaks were observed. One of them was formed via reaction 2 (K_0_ = 1.52 cm^2^·V^−1^·s^−1^), and the other two peaks with reduced ion mobility of 1.42 and 1.34 cm^2^·V^−1^·s^−1^ were apparently created by ionization with N_x_O_y_ [51]. Please see the Supplementary Materials for the full set of IMS spectra of DNT isomers.

The direct detection of explosives using the LD-IMS technique was possible with almost all investigated surfaces (aluminum, stainless steel, ceramic, PVC, glass, drywall, and paper). For highly reflective materials such as metals and ceramics, the detection efficiency was significantly reduced due to the back-reflection of laser light, which hindered effective surface heating. Similarly, transparent materials like glass exhibited lower desorption efficiency due to light penetration through the material. Explosives such as RDX/C-4, PETN/Semtex, and DNTs were particularly difficult to detect on these surfaces. To counter these challenges, darkening the sample area with a black marker greatly enhanced the desorption efficiency and improved the reproducibility of the results. For example, in the case of TNT, the detection sensitivity increased approximately tenfold after surface darkening. This improvement underscores the necessity of darkening for reliable detection on reflective and transparent surfaces. After using the marker, it was important to allow enough time (around 1 min) for the evaporation of the solvents from the marker ink; otherwise, a peak with a reduced mobility of 1.63 cm^2^·V^−1^·s^−1^ was present in the spectrum (Figure 2d) (see the Supplementary Materials, Figure S9.1). 

The LD technique has its limitations in the case of flammable materials or materials with low melting points. For such materials, LD can result in the unwanted destruction or desorption of the surface material. In the case of PVC, after focusing the laser on the PVC plate, the material started to melt and smoke. The IMS response to the PVC resulted in the formation of a peak with a reduced ion mobility of 1.06 cm^2^·V^−1^·s^−1^, as shown in Figure 2c. The position of this peak is outside the region where peaks from the explosives typically appear and does not impose any limitation on their detection. Different results were obtained from other flammable materials such as paper, wood, and textiles. These materials ignited rapidly, greatly limiting or preventing any detection of explosives. Figure 2f,g show the IMS spectra of PETN and RDX detected from paper. Despite the broad structure in the IMS spectrum, visible peaks of PETN/Semtex and RDX/C-4 were detected. The LD-IMS detection of explosives in the flammable materials wood, denim, and cotton was impossible (Figure 2h). For these materials, it is more appropriate to use a different, less invasive method, such as thermal desorption [19,27,47,48]. 

### 2.2. Limit of Detection

**Figure 3:**
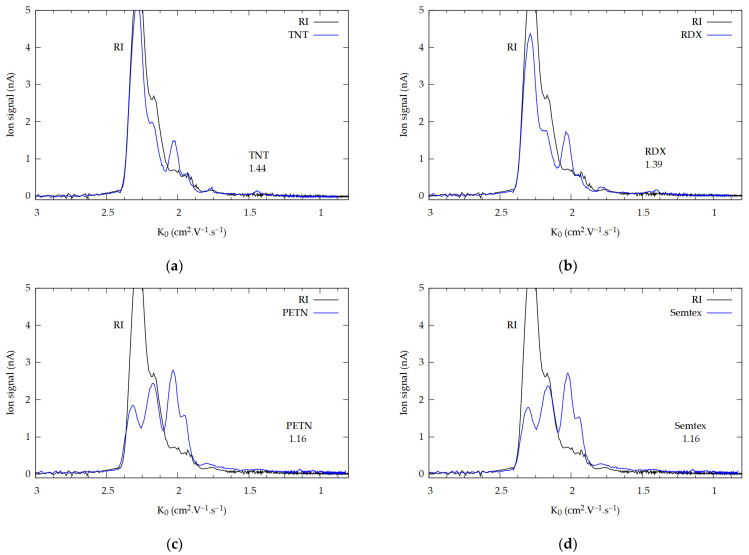
Measured sensitivity for (**a**) 50 ng (7 ng/mm^2^) of TNT, (**b**) 100 ng (15 ng/mm^2^) of RDX, (**c**) 100 ng (15 ng/mm^2^) of PETN, and (**d**) 100 ng (15 ng/mm^2^) of Semtex, detected from ceramic.

Another important aspect of the present study was determining the limit of detection (LOD) for explosives on these surfaces. For this study, samples were prepared by depositing 50 ng of TNT, 100 ng of RDX, and 100 ng of PETN on PVC. The detailed procedure of sample preparation for LOD measurements is described in Section 3.3. By converting the surface densities of the samples to the irradiated spot, the LODs were calculated: 7 ng/mm^2^ for TNT and 15 ng/mm^2^ for RDX/C-4 and PETN/Semtex. The corresponding spectra are presented in Figure 3a–d, respectively. Drywall, a porous and thermally insulating material, exhibited weaker heating, which led to reduced desorption efficiency of the explosive from its surface. In contrast, the remaining materials (after darkening) demonstrated more effective thermal conductivity, resulting in more efficient surface heating and enhanced desorption of the explosive compared to drywall. In the case of 2,4-DNT, 3,4-DNT, and 2,6-DNT, the LODs were calculated to be 50, 80, and 80 ng/mm^2^. 

These LODs were determined for aluminum, stainless steel, ceramic, PVC, and glass. Interestingly, desorption from the drywall surface exhibited a weaker response (approximately by a factor of 2), resulting in a lower detection limit. For TNT, the detection limit was 15 ng/mm^2^; for RDX/C-4 and PETN/Semtex, it was 30 ng/mm^2^; for 2,4-DNT, it was 80 ng/mm^2^; and for 3,4-DNT and 2,6-DNT, it increased to 200 ng/mm^2^. The darkening of the surface with the marker caused the materials to acquire the same ability to absorb thermal energy from the laser, which probably achieved an equalizing effect where the same amount of substance was desorbed from different surfaces. This process ensures that the LODs are almost the same for all materials. Overall, the desorption of substances from the surface depends on the structure of the material (drywall) as well as the color. A summary of all LODs is shown in Table 1.

The present LOD results can be compared with an earlier study by Ilbeigi et al. [21]. They detected TNT, RDX, and PETN using LD-IMS from thin-layer chromatography (TLC) plates. The TLC plates consisted of a silica gel matrix coated on alumina plates. They reported an LOD for TNT of 30 ng and 80 ng for RDX and PETN [21]. The IMS conditions used in their study were comparable to ours. 

Akmalov et al. [52] researched the detection of explosives (TNT, RDX, PETN, and HMX) on different surfaces, including quartz glass, aluminum, paper, and polyethylene. They demonstrated that the required time to achieve distinct desorption results ranged from 2 to 10 s. In their study, a Nd^3+^:YAG laser was employed for the desorption of explosives, with a pulse duration of 6 ns. The frequency of irradiation increased the quantity of desorbed matter, not only by increasing the number of pulses but also by providing additional substrate warming. The typical amount of desorbed matter ranged from 95 ng to 7900 ng [52]. 

Ehlert et al. [20] studied LD-IMS detection from aluminum foil and obtained LODs of 1 ng for TNT, 25 ng for RDX, and 10 ng for PETN, which are comparable to our calculated LODs.

## 3. Materials and Methods

### 3.1. Experimental Setup

**Figure 4:**
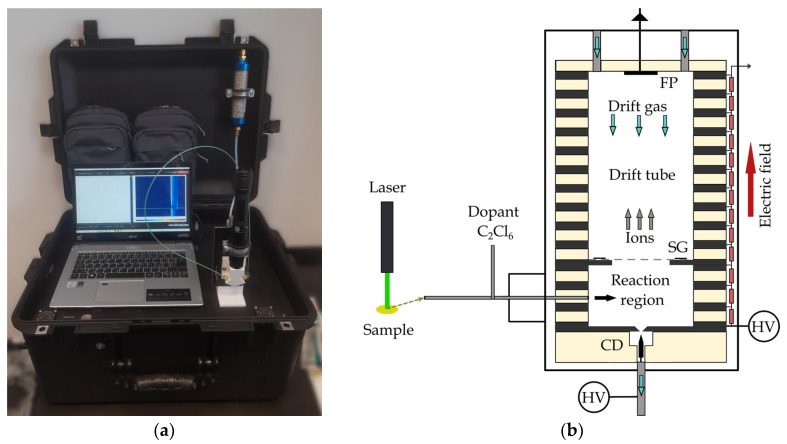
(**a**) Storage of the IMS instrument in a transport box with electronics, battery, flow system, and laser; (**b**) scheme of IMS with dopant gas C_2_Cl_6_ and laser.

The ion mobility spectrometry (IMS) system used in this study to detect explosives was developed by MaSa Tech Ltd., Company (Stará Turá, Slovakia). The IMS device was boxed with all electronics, power supplies, filters, and dopant gas (Figure 4a). The device was powered by a battery that allowed continuous operation for up to 8 h. This makes the IMS a highly portable analytical device. The IMS instrument was constructed using multiple stainless-steel ring electrodes isolated by Teflon rings, resulting in a total length of the drift tube of 11.16 cm, as shown in Figure 4b. The corona discharge (CD) ionization source was operated in negative polarity, following a point-to-plane geometry, with the gas outlet positioned behind the discharge, allowing the CD to operate in a reverse-flow regime. For IMS equipment, a Bradbury–Nielsen-type shutter grid (SG) with an opening time of 150 µs and a period of 14,500 µs was employed. A potential difference of 3.6 kV was applied across the CD, and the electric field intensity inside the drift tube was set to 509.8 V.cm^−1^. As a drift gas was used, purified atmospheric air was obtained through a zeolite filter (Agilent Technologies, Inc., Santa Clara, CA, USA) with a typical flow rate of 600 mL/min. The operational pressure was maintained at 600 mbar, and the IMS drift tube temperature was set to 60 °C. The desorbed sample was sucked through a 50 cm long PEEK capillary with an inner diameter of 0.8 mm. The sample gas flow comprised 500 mL/min of atmospheric air without additional purification but with an admixture of C_2_Cl_6_ dopant with a concentration of 750 ppb. The capillary input was positioned perpendicular to the laser beam, several millimeters from the sample surface. A standard diode laser operating at a wavelength of 532 nm (green light) with a power of 1000 mW was employed in the setup. The spot of the focused laser beam was 1.4 mm^2^. The irradiation time was exactly one second for all measurements.

### 3.2. Chemicals and Materials

Hexachloroethane (C_2_Cl_6_) (Sigma-Aldrich, Burlington, MA, USA) of 99% purity was used as a dopant gas. The explosives 2,4,6-Trinitrotoluene (TNT), Cyclotrimethylenetrinitramine (RDX), pentaerythritol tetranitrate (PETN), Composition C-4 (contains 91% of RDX), Semtex A1 (contains 83% of PETN), 2,4-Dinitrotoluene (2,4-DNT), 3,4-Dinitrotoluene (3,4-DNT), and 2,6-Dinitrotoluene (2,6-DNT) were obtained from the Slovak Department of Defence with a purity of up to 99%. All compounds are shown in Table 2. 

**Figure 5:**
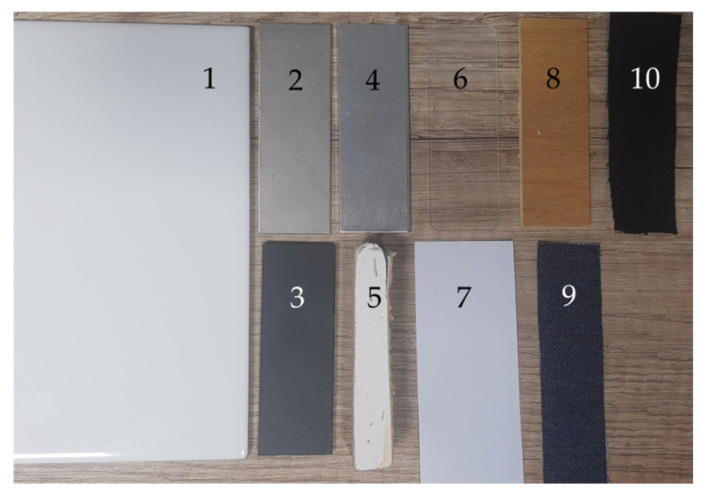
Image of various surfaces used in this study: 1—ceramic; 2—stainless steel; 3—PVC; 4—aluminum; 5—drywall; 6—glass; 7—paper; 8—wood; 9—denim; 10—cotton.

Explosive detection was performed on typical surfaces to be found in the real world, including aluminum, stainless steel, ceramic, PVC, glass, drywall, paper, wood, cotton, and denim, as presented in Figure 5. All materials were obtained from the Netherlands Forensic Institute (NFI) as part of the RISEN project (real-time on-site forensic trace qualification).

### 3.3. Sample Preparation

The TNT and DNT compounds were diluted in analytical-grade methanol, while RDX, C-4, PETN, and Semtex were diluted in analytical-grade acetone (Sigma-Aldrich). These solutions were prepared at a concentration of 1 mg/mL. The explosives were weighed on an analytical balance (KERN Ltd., London, UK), and solvents were added via 1 mL injection (B. Braun Medical, Melsungen, Germany). A 10 µL syringe (Hamilton Company, Reno, NV, USA) was used to deposit the solution onto the swipe material being tested. The application of the sample was carried out in several small drops while waiting for the solvent to evaporate, thus ensuring the sample spot at 20–25 mm^2^. The area of the spot was calculated from the diameter of the spot.

For LOD measurements, solutions of explosives with a concentration of 100 µg/mL were prepared, and a 1 µL syringe (Hamilton Company) was used to apply the samples to the surface of the materials. In this case, 100 ng (full syringe) and 50 ng (half syringe) were deposited on the surface. The desorbed amount was calculated as the ratio of the desorbed area (laser spot) and sample spot area. The sample spot size was typically distributed over the surface area of 10 mm^2^. Detailed values of the detection limits for each explosive are clarified in Section 2.2. To solve the problem of laser reflection from highly reflective materials such as metals and ceramics and light penetration into transparent materials such as glass, a black marker (Centropen, as, Dačice, Czech Republic) was used to darken the surface. The marker was applied only after depositing the sample on the material’s surface, representing the only effect usable even in real conditions. A waiting period was observed after marker application to ensure the complete evaporation of the solvents from the ink markers, which represents potential interference with the detection signals. In order to avoid biasing the results and reducing the intensity of the signals, it was necessary to wait approximately 1 min for all solvents present in the ink to evaporate.

## 4. Conclusions

The current study illustrates the successful implementation of ion mobility spectrometry (IMS) in conjunction with laser desorption (LD) for detecting explosives on various surfaces, such as aluminum, stainless steel, ceramic, PVC, glass, drywall, paper, wood, cotton, and denim. This detection approach utilized IMS with a corona discharge (CD) ion source and C_2_Cl_6_ dopant. LD emerged as an efficient sampling technique for desorbing low-volatility compounds, such as explosives, enabling their subsequent analysis through IMS. 

The results highlight the influence of surface materials on LD-IMS performance. Explosive samples were detectable on nearly all surfaces, except for flammable materials like paper, wood, and textiles, which ignited when subjected to laser focusing. PVC and drywall surfaces exhibited favorable responses, while challenges arose with ceramic and metal materials due to laser reflection. We ensured successful desorption by darkening the area where the sample was applied.

The reduced ion mobilities were as follows: TNT, 1.44 cm^2^·V^−1^·s^−1^; RDX and C-4, 1.39 cm^2^·V^−1^·s^−1^; PETN and Semtex, 1.16 cm^2^·V^−1^·s^−1^; 2,4-DNT, 1.55 cm^2^·V^−1^·s^−1^; 3,4-DNT, 1.52 cm^2^·V^−1^·s^−1^; and 2,6-DNT, 1.47 cm^2^·V^−1^·s^−1^. Furthermore, the LODs for each explosive were determined when subjected to LD on aluminum, stainless steel, ceramic, PVC, and paper. The corresponding LOD values were 7 ng/mm^2^ for TNT; 15 ng/mm^2^ for RDX, C-4, PETN, and Semtex; and approximately 50 ng/mm^2^ for DNTs. Importantly, drywall yielded slightly lower sensitivity for all explosive samples.

## Figures and Tables

**Table 1:** List of the determined LODs of explosives in ng/mm^2^.

Sample	Alu	SS	Ceramic	PVC	Glass	Drywall	Paper	Wood	Cotton	Denim
TNT	7	7	7	7	7	15	nd	nd	nd	nd
RDX	15	15	15	15	15	30	nd *	nd	nd	nd
PETN	15	15	15	15	15	30	nd *	nd	nd	nd
C-4	15	15	15	15	15	30	nd *	nd	nd	nd
Semtex	15	15	15	15	15	30	nd *	nd	nd	nd
2,4-DNT	50	50	50	50	50	80	nd	nd	nd	nd
3,4-DNT	80	80	80	80	80	200	nd	nd	nd	nd
2,6-DNT	80	80	80	80	80	200	nd	nd	nd	nd

nd—not detected. * Detection is possible, but the LOD has not been determined.

**Table 2:** List of used explosives with CAS numbers.

Explosives	CAS Number
TNT	118-96-7
RDX	121-82-4
PETN	78-11-5
C-4	
Semtex	
2,4-DNT	121-14-2
3,4-DNT	610-39-9
2,6-DNT	606-20-2

## Data Availability

The data are part of the article and supplementary materials.

## Supplementary Materials

The following supporting information can be downloaded at: https://www.mdpi.com/article/10.3390/molecules29184482/s1. Figure S1.1: The IMS spectrum of TNT from an aluminum plate. Figure S1.2: The IMS spectrum of RDX from aluminum. Figure S1.3: The IMS spectrum of PETN from aluminum. Figure S1.4: The IMS spectrum of C-4 from aluminum. Figure S1.5: The IMS spectrum of Semtex from aluminum. Figure S1.6: The IMS spectrum of 2,4-DNT from aluminum. Figure S1.7: The IMS spectrum of 3,4-DNT from aluminum. Figure S1.8: The IMS spectrum of 2,6-DNT from aluminum. Figure S2.1: The IMS spectrum of TNT from ceramic. Figure S2.2: The IMS spectrum of RDX from ceramic. Figure S2.3: The IMS spectrum of PETN from ceramic. Figure S2.4: The IMS spectrum of C-4 from ceramic. Figure S2.5: The IMS spectrum of Semtex from ceramic. Figure S2.6: The IMS spectrum of 2,4-DNT from ceramic. Figure S2.7: The IMS spectrum of 3,4-DNT from ceramic. Figure S2.8: The IMS spectrum of 2,6-DNT from ceramic Figure S3.1: The IMS spectrum of TNT from drywall. Figure S3.2: The IMS spectrum of RDX from drywall. Figure S3.3: The IMS spectrum of PETN from drywall. Figure S3.4: The IMS spectrum of C-4 from drywall. Figure S3.5: The IMS spectrum of Semtex from drywall. Figure S3.6: The IMS spectrum of 2,4-DNT from drywall. Figure S3.7: The IMS spectrum of 3,4-DNT from drywall. Figure S3.8: The IMS spectrum of 2,6-DNT from drywall. Figure S4.1: The IMS spectrum of TNT from glass. Figure S4.2: The IMS spectrum of RDX from glass. Figure S4.3: The IMS spectrum of PETN from glass. Figure S4.4: The IMS spectrum of C-4 from glass. Figure S4.5: The IMS spectrum of Semtex from glass. Figure S4.6: The IMS spectrum of 2,4-DNT from glass. Figure S4.7: The IMS spectrum of 3,4-DNT from glass. Figure S4.8: The IMS spectrum of 2,6-DNT from glass. Figure S5.1: The IMS spectrum of TNT from PVC. Figure S5.2: The IMS spectrum of RDX from PVC. Figure S5.3: The IMS spectrum of PETN from PVC. Figure S5.4: The IMS spectrum of C-4 from PVC. Figure S5.5: The IMS spectrum of Semtex from PVC. Figure S5.6: The IMS spectrum of 2,4-DNT from PVC. Figure S5.7: The IMS spectrum of 3,4-DNT from PVC. Figure S5.8: The IMS spectrum of 2,6-DNT from PVC. Figure S6.1: The IMS spectrum of TNT from stainless-steel. Figure S6.2: The IMS spectrum of RDX from stainless-steel. Figure S6.3: The IMS spectrum of PETN from stainless-steel. Figure S6.4: The IMS spectrum of C-4 from stainless-steel. Figure S6.5: The IMS spectrum of Semtex from stainless-steel. Figure S6.6: The IMS spectrum of 2,4-DNT from stainless-steel. Figure S6.7: The IMS spectrum of 3,4-DNT from stainless-steel. Figure S6.8: The IMS spectrum of 2,6-DNT from stainless-steel. Figure S7.1: The IMS spectrum of TNT from paper. Figure S7.2: The IMS spectrum of RDX from paper. Figure S7.3: The IMS spectrum of PETN from paper. Figure S7.4: The IMS spectrum of C-4 from paper. Figure S7.5: The IMS spectrum of Semtex from paper. Figure S8.1: The IMS spectrum of PETN from wood. Figure S9.1: The IMS spectrum of the marker (Centropen).
