# Supplementary material for: Laser Desorption of Explosives from the Surface of Different Real-World Materials Studied Using C2Cl6-Dopant-Assisted Ion Mobility Spectrometry
Source: Molecules. 2024 Sep 21;29(18):4482. doi: 10.3390/molecules29184482 (PMC11433934; doi:10.3390/molecules29184482)
Supplement: Supplementary file 1 [file molecules-29-04482-s001.zip › molecules-3172287-supplementary.pdf]

Supplementary materials to the article

Laser Desorption of Explosives from the Surface of Different Real-World Materials Studied Using C_2Cl_6 Dopant Assisted Ion Mobility Spectrometry

General remarks:

The studied spectra are divided into eight sections according to the examined materials—aluminum, ceramics, drywall, glass, PVC, stainless steel, paper, wood—and one extra section for the marker. Each section (excluding paper and wood) consists of the spectra of all eight investigated explosives. The black curve in the IMS spectra represents the spectrum of the reactant ions, the blue curve represents the blank spectrum of the examined material, and the red curve represents the spectrum of the investigated material with the explosive.

The reduced ion mobilities:

THT = 1.44 $cm^2.V^{-1}.s^{-1}$

RDX = 1.39 $cm^2.V^{-1}.s^{-1}$

PETN = 1.16 $cm^2.V^{-1}.s^{-1}$

C-4 = 1.39 $cm^2.V^{-1}.s^{-1}$

Semtex = 1.16 $cm^2.V^{-1}.s^{-1}$

2,4-DNT = 1.55 $cm^2.V^{-1}.s^{-1}$

3,4-DNT = 1.52; 1.42; 1.34 $cm^2.V^{-1}.s^{-1}$

2,6-DNT = 1.48 $cm^2.V^{-1}.s^{-1}$

Marker = 1.63 $cm^2.V^{-1}.s^{-1}$

A marker (Centropen) was used to increase the light absorption by the surface. The ion peak of the marker is only visible in some IMS spectra. In these cases, the waiting time between marker application and measurement of the spectrum was short, and the solvent from the marker was still present. A typical IMS spectrum of the marker is presented in Fig. 9.1.

Section 1. Aluminum

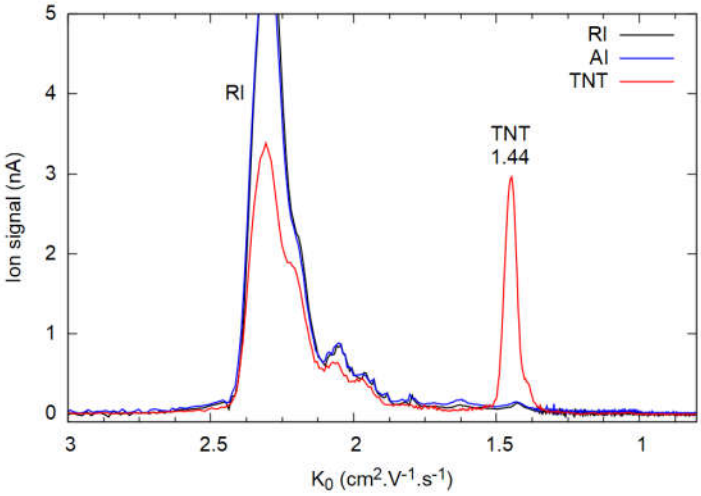

Figure S1.1: The IMS spectrum of TNT from aluminum.

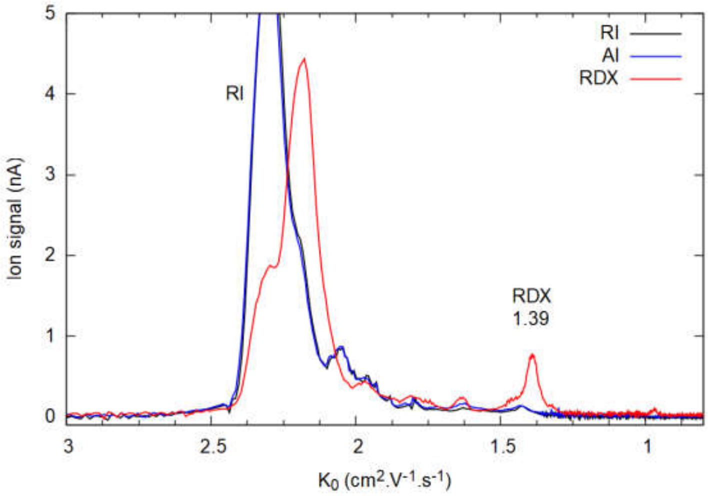

Figure S1.2: The IMS spectrum of RDX from aluminum.

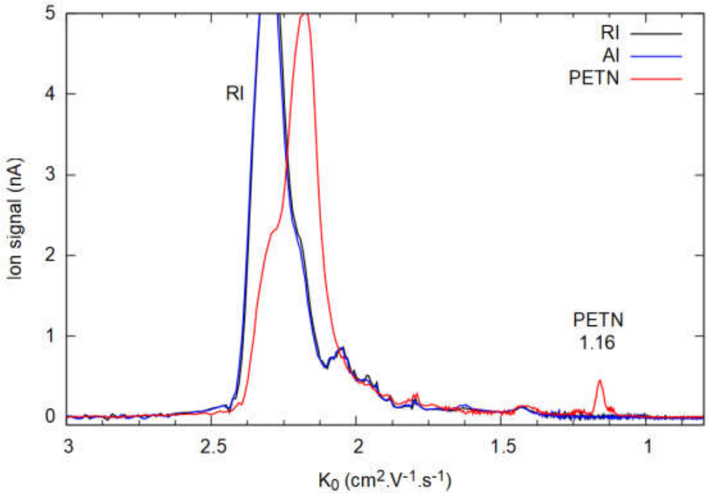

Figure S1.3: The IMS spectrum of PETN from aluminum.

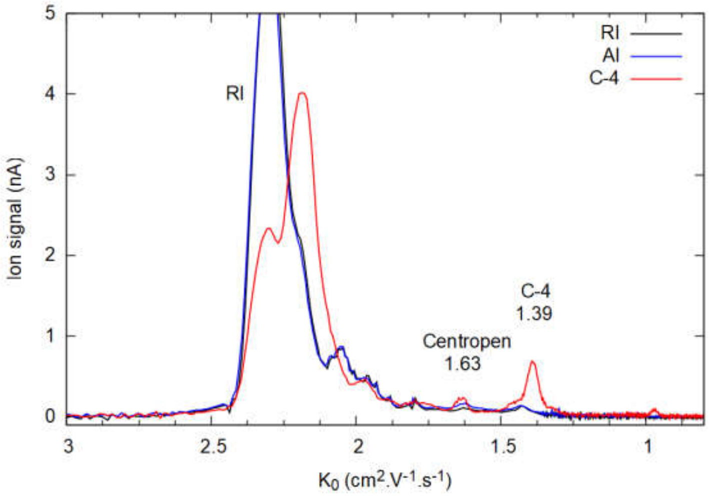

Figure S1.4: The IMS spectrum of C-4 from aluminum.

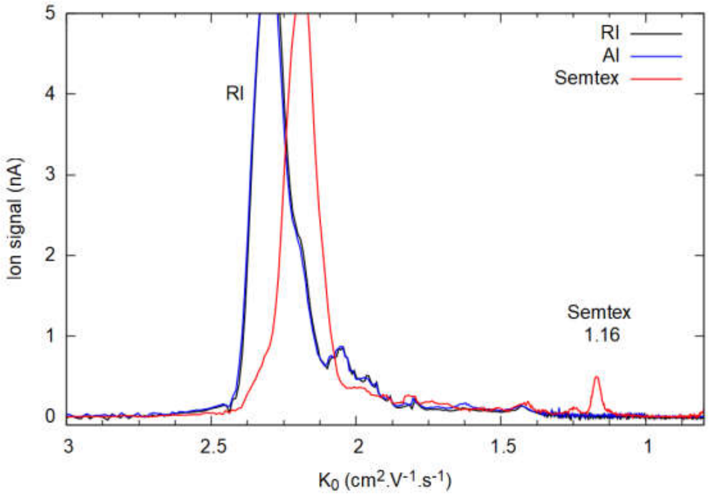

Figure S1.5: The IMS spectrum of Semtex from aluminum.

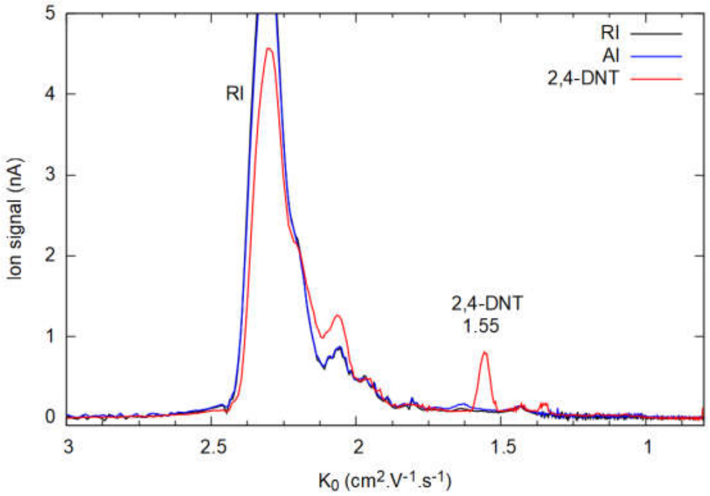

Figure S1.6: The IMS spectrum of 2,4-DNT from aluminum.

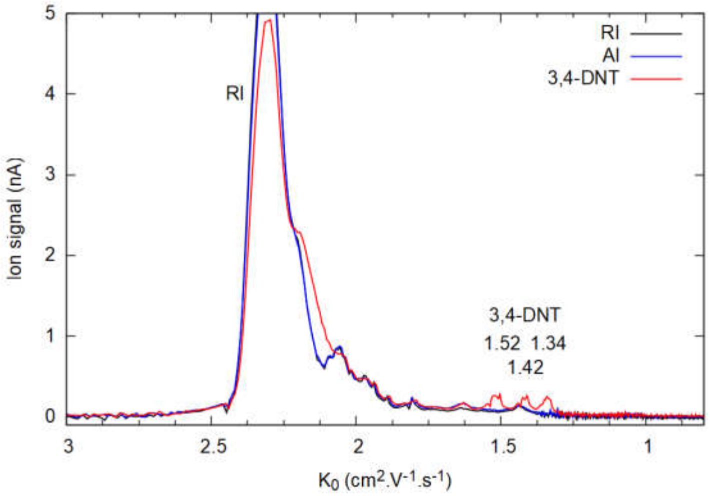

Figure S1.7: The IMS spectrum of 3,4-DNT from aluminum.

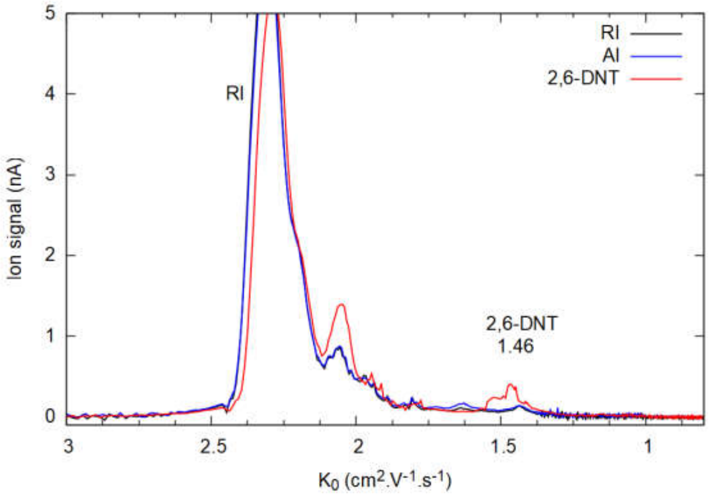

Figure S1.8: The IMS spectrum of 2,6-DNT from aluminum.

Section 2. Ceramic

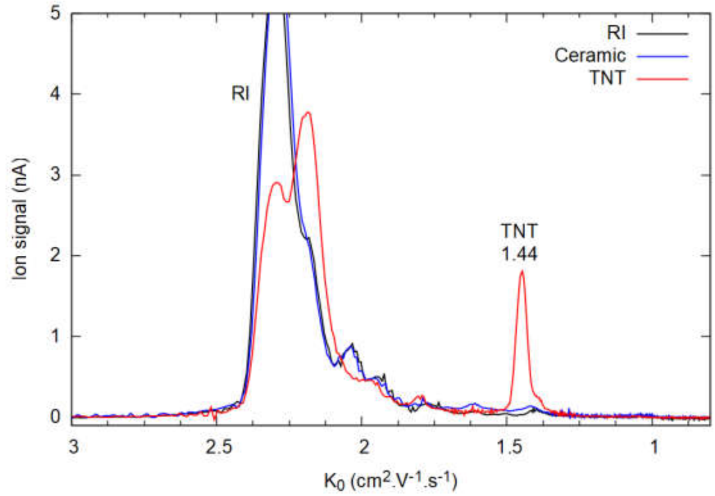

Figure S2.1: The IMS spectrum of TNT from ceramic.

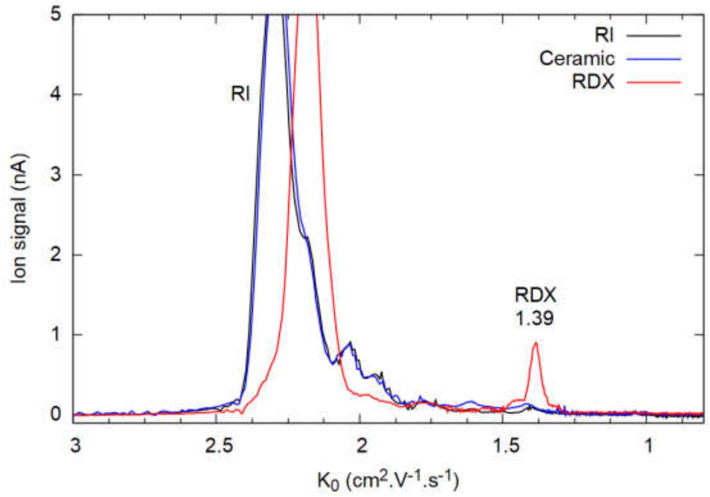

Figure S2.2: The IMS spectrum of RDX from ceramic.

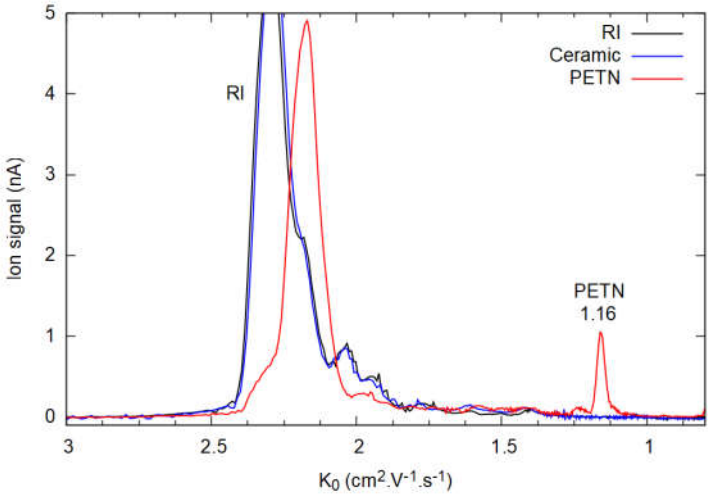

Figure S2.3: The IMS spectrum of PETN from ceramic.

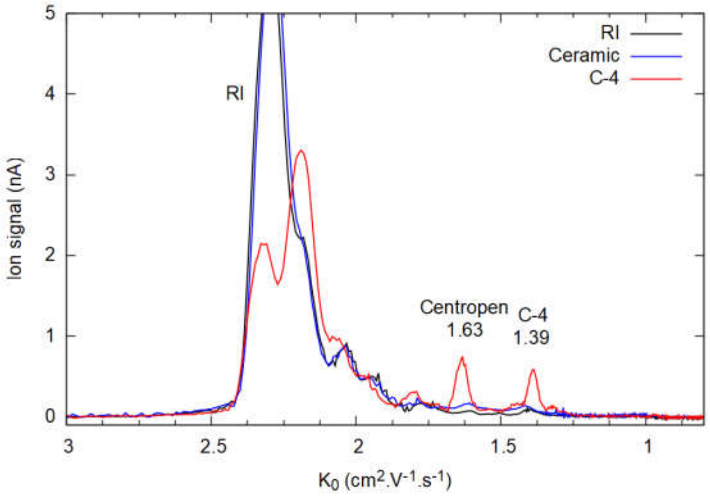

Figure S2.4: The IMS spectrum of C-4 from ceramic.

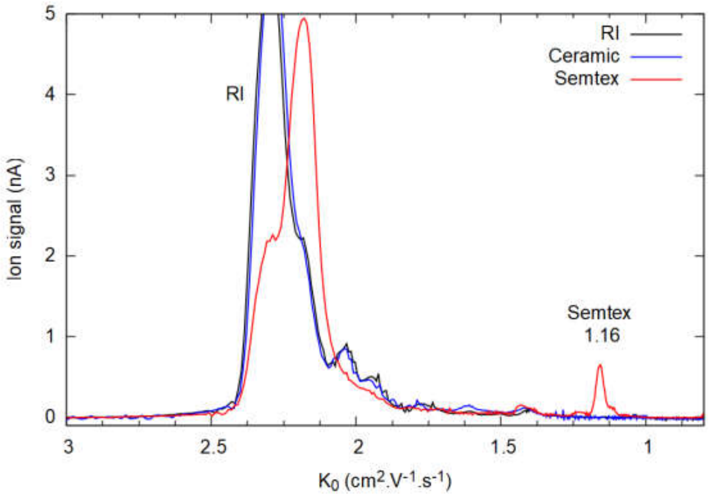

Figure S2.5: The IMS spectrum of Semtex from ceramic.

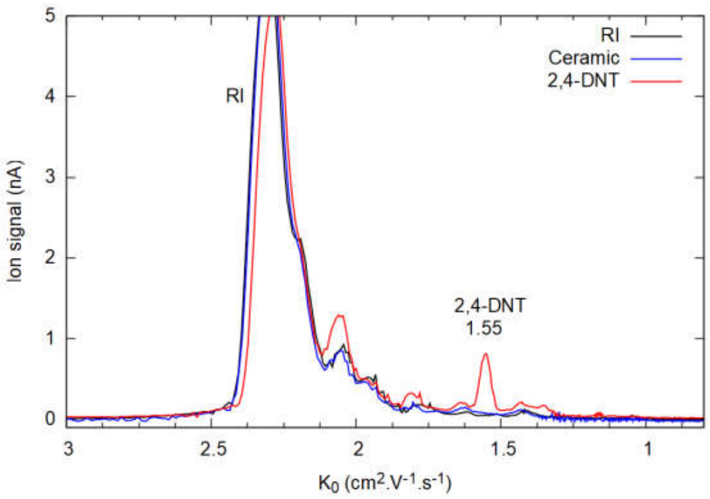

Figure S2.6: The IMS spectrum of a 2,4-DNT from a Ceramic

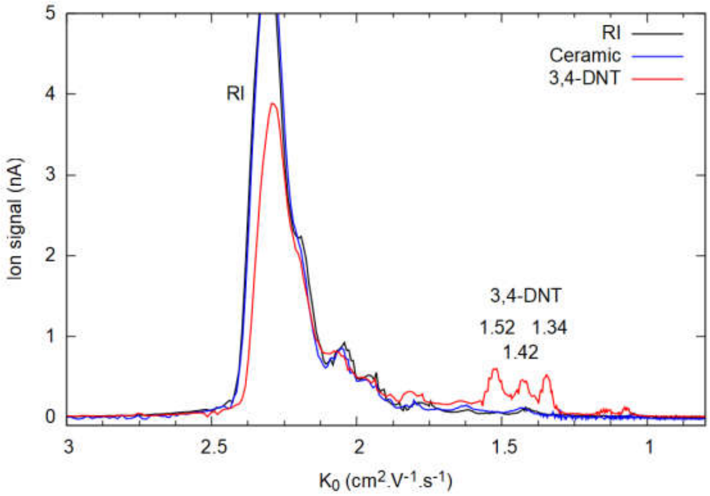

Figure S2.7: The IMS spectrum of 3,4-DNT from a Ceramic

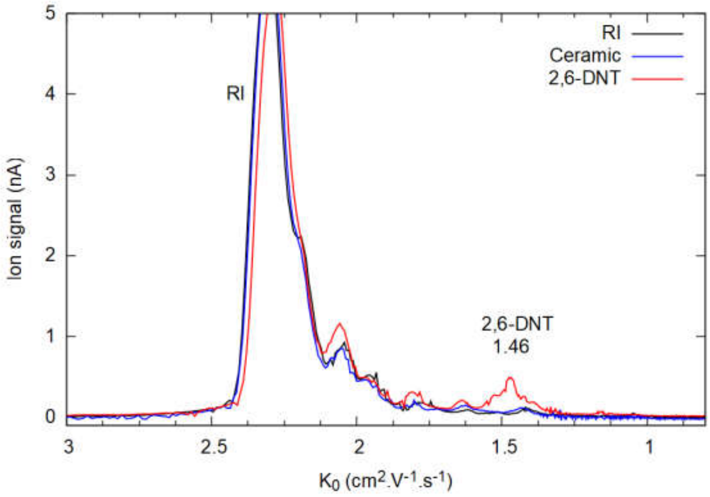

Figure S2.8: The IMS spectrum of 2,6-DNT from a Ceramic

Section 3. Drywall

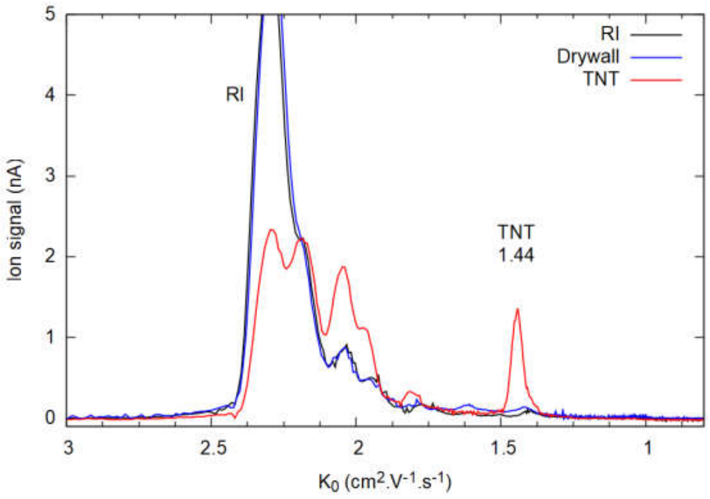

Figure S3.1: The IMS spectrum of TNT from drywall.

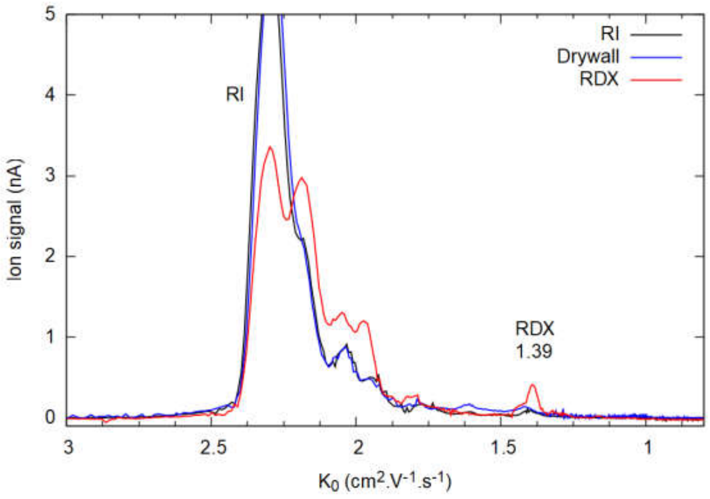

Figure S3.2: The IMS spectrum of RDX from drywall.

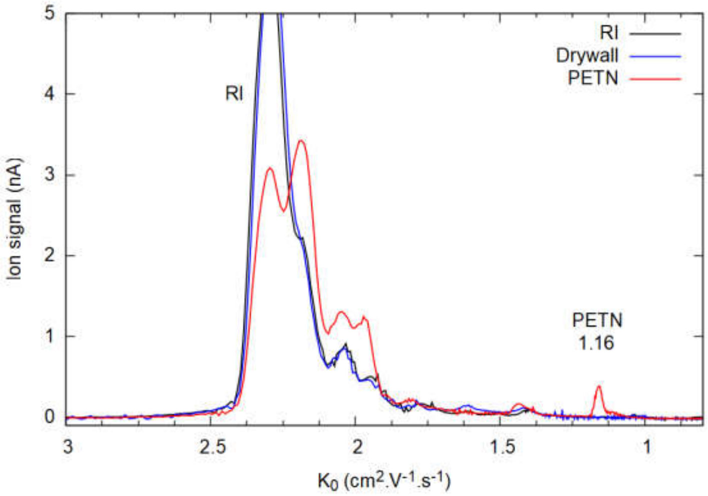

Figure S3.3: The IMS spectrum of PETN from drywall.

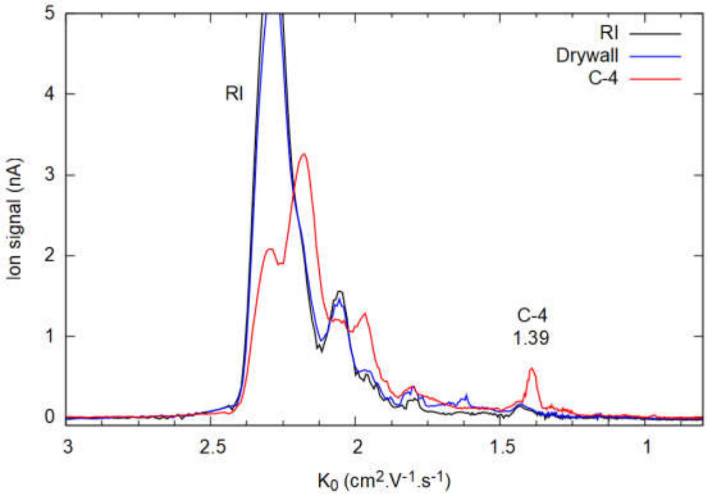

Figure S3.4: The IMS spectrum of C-4 from drywall.

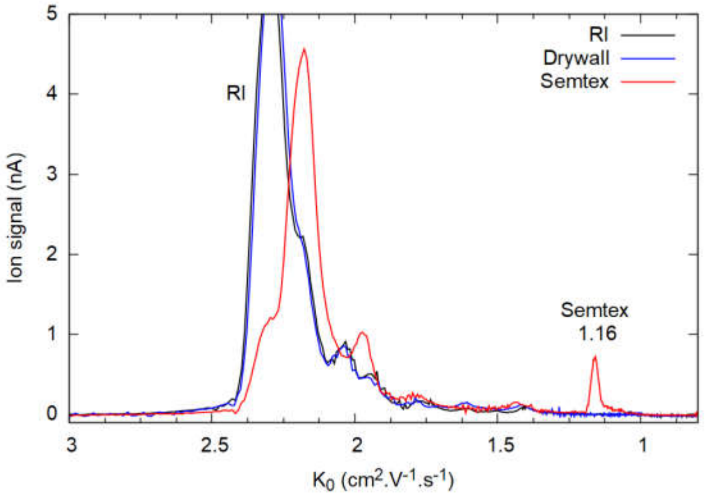

Figure S3.5: The IMS spectrum of Semtex from drywall.

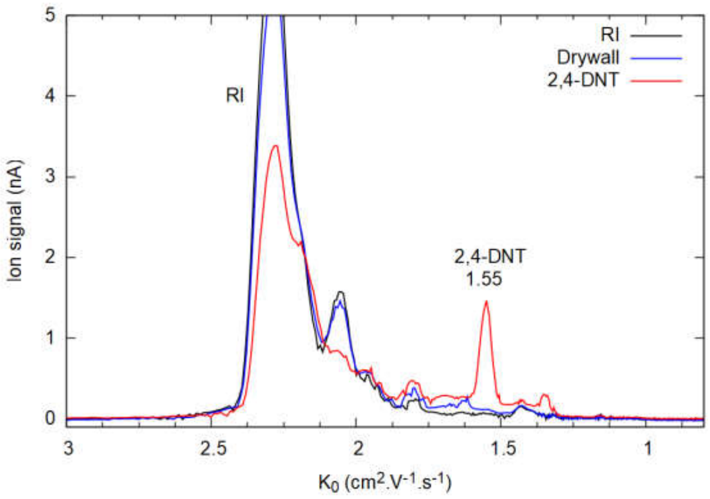

Figure S3.6: The IMS spectrum of 2,4-DNT from drywall.

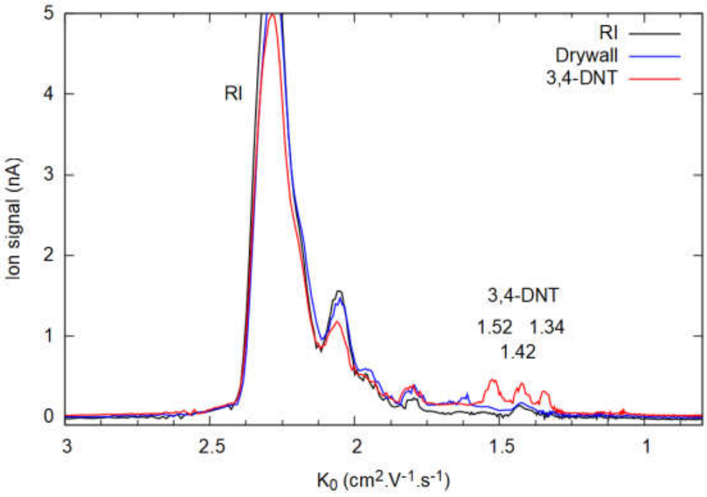

Figure S3.7: The IMS spectrum of 3,4-DNT from drywall.

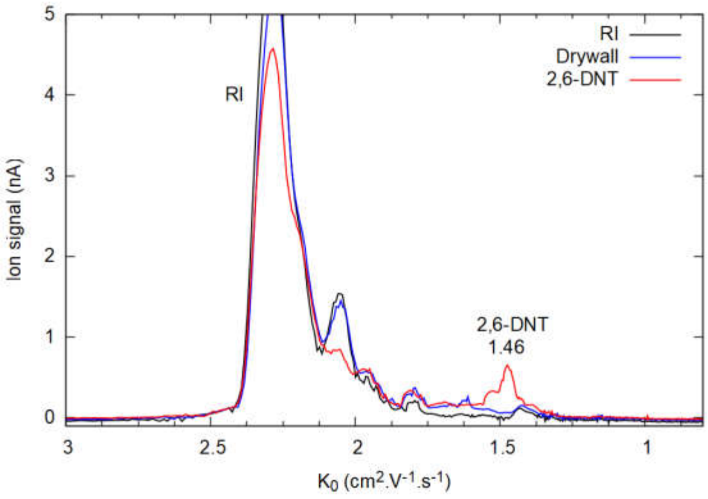

Figure S3.8: The IMS spectrum of 2,6-DNT from drywall.

Section 4. Glass

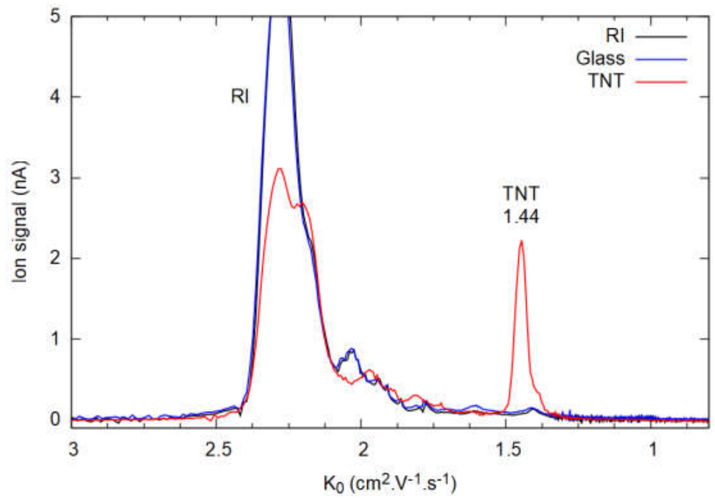

Figure S4.1: The IMS spectrum of TNT from glass.

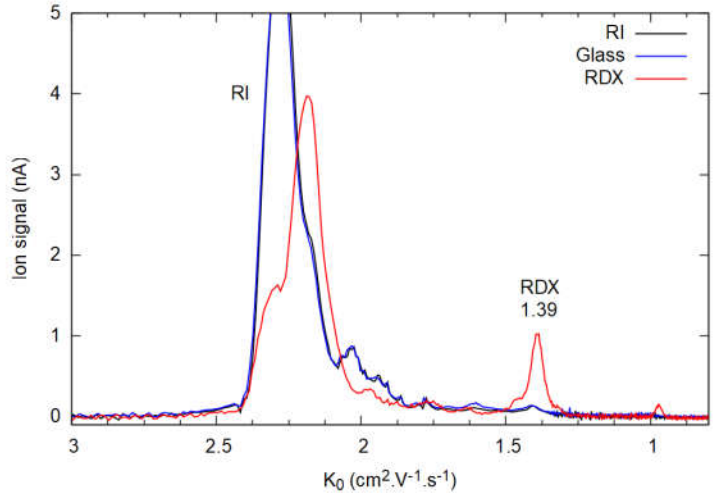

Figure S4.2: The IMS spectrum of RDX from glass.

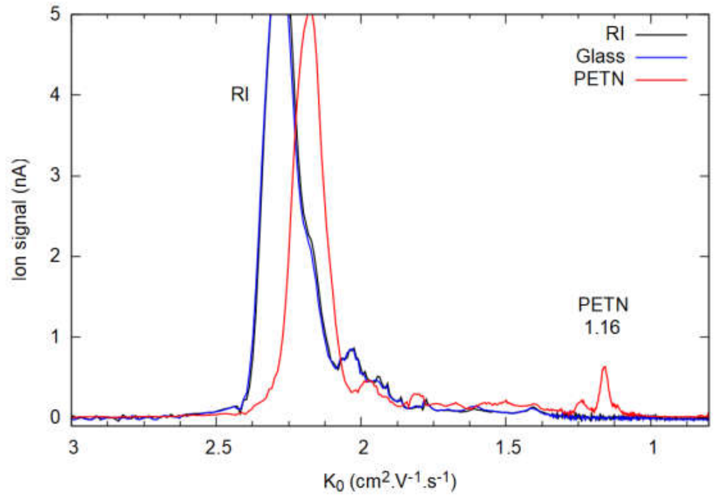

Figure S4.3: The IMS spectrum of PETN from glass.

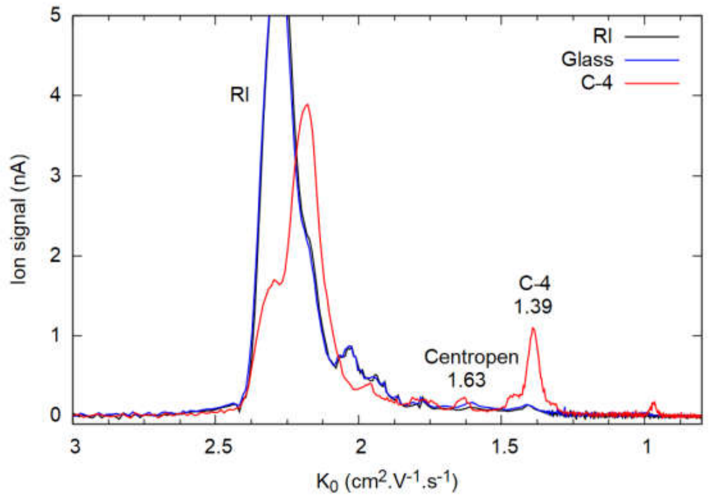

Figure S4.4: The IMS spectrum of C-4 from glass.

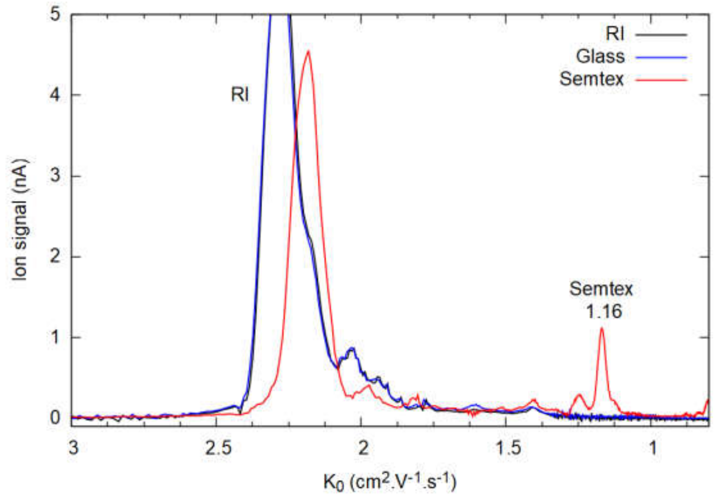

Figure S4.5: The IMS spectrum of Semtex from glass.

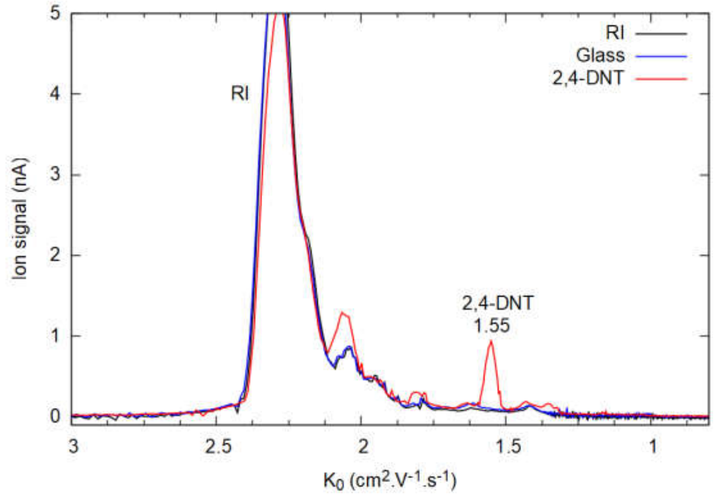

Figure S4.6: The IMS spectrum of 2,4-DNT from glass.

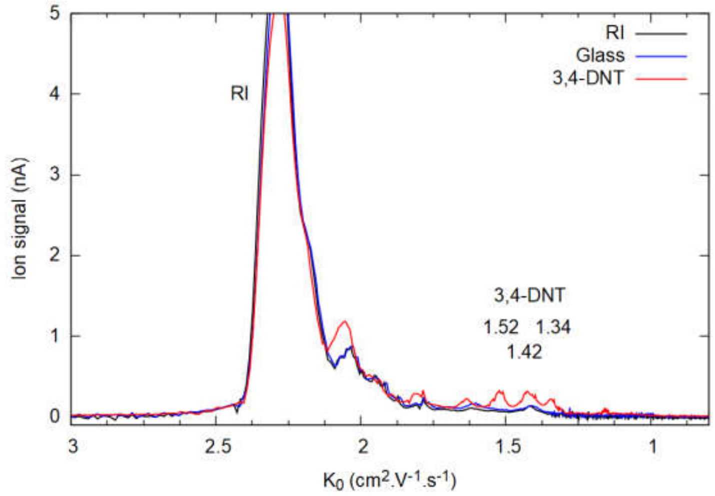

Figure S4.7: The IMS spectrum of 3,4-DNT from glass.

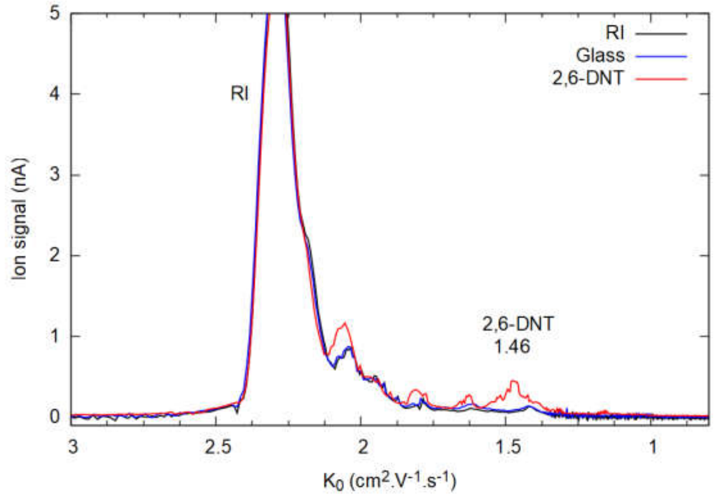

Figure S4.8: The IMS spectrum of 2,6-DNT from glass.

Section 5. PVC

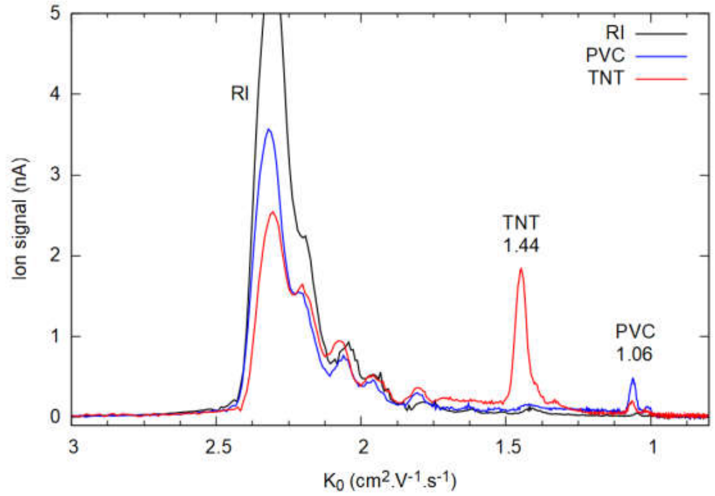

Figure S5.1: The IMS spectrum of TNT from PVC.

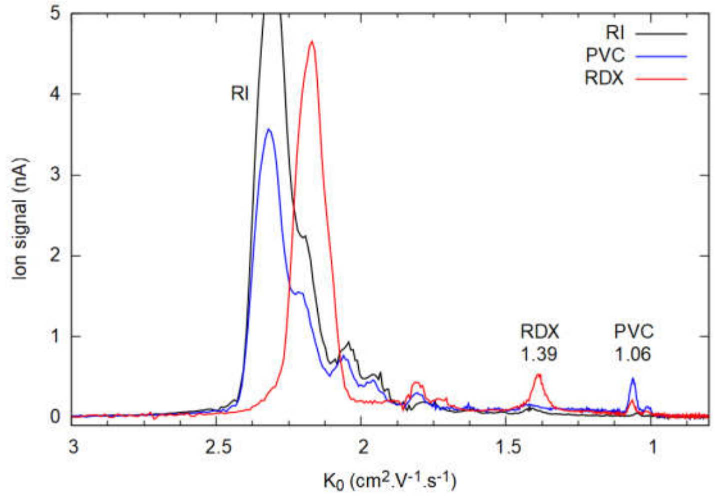

Figure S5.2: The IMS spectrum of RDX from PVC.

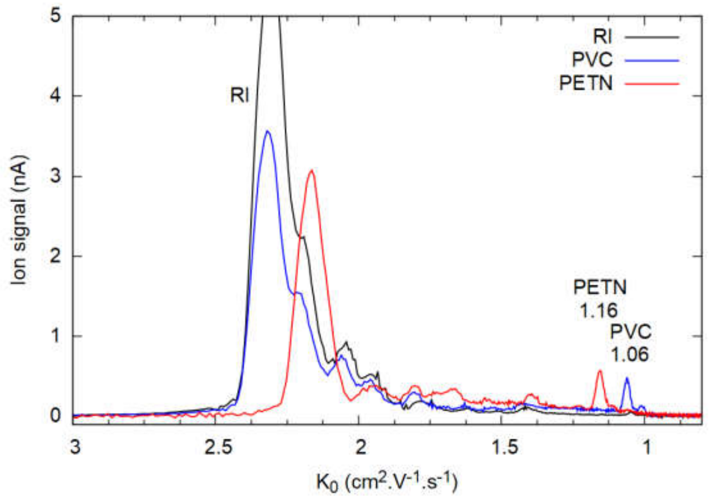

Figure S5.3: The IMS spectrum of PETN from PVC.

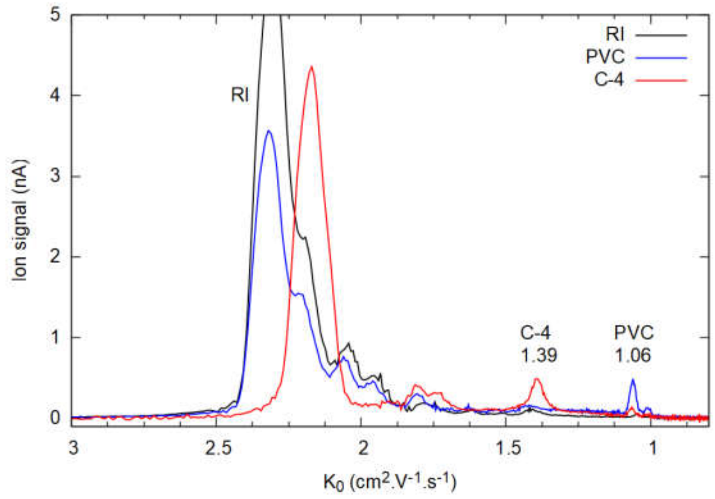

Figure S5.4: The IMS spectrum of C-4 from PVC.

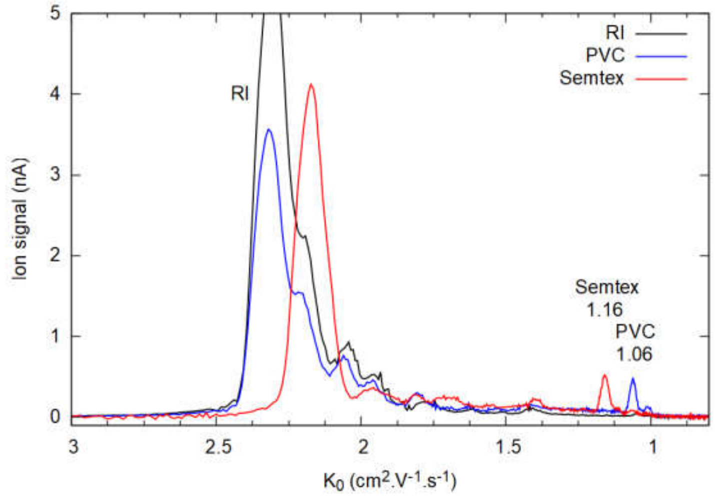

Figure S5.5: The IMS spectrum of Semtex from PVC.

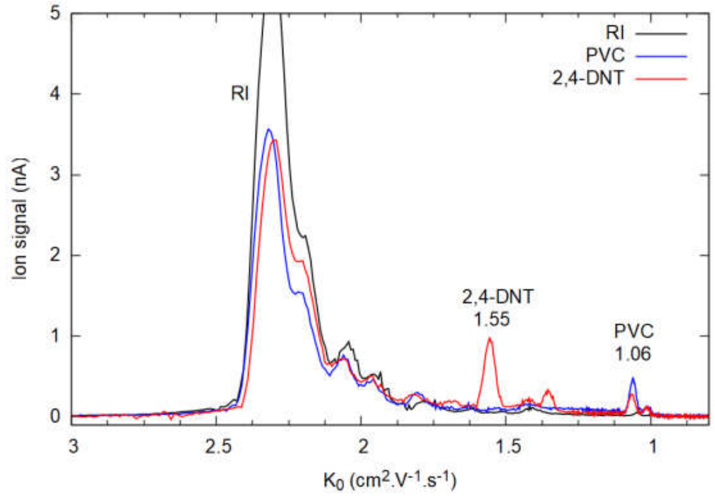

Figure S5.6: The IMS spectrum of 2,4-DNT from PVC.

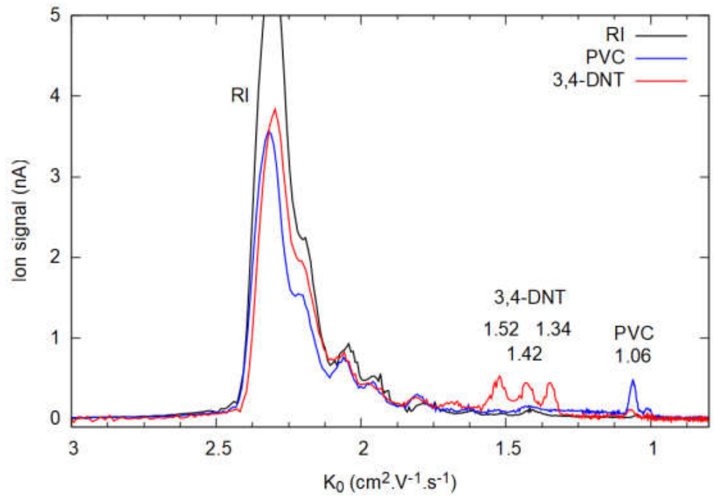

Figure S5.7: The IMS spectrum of 3,4-DNT from PVC.

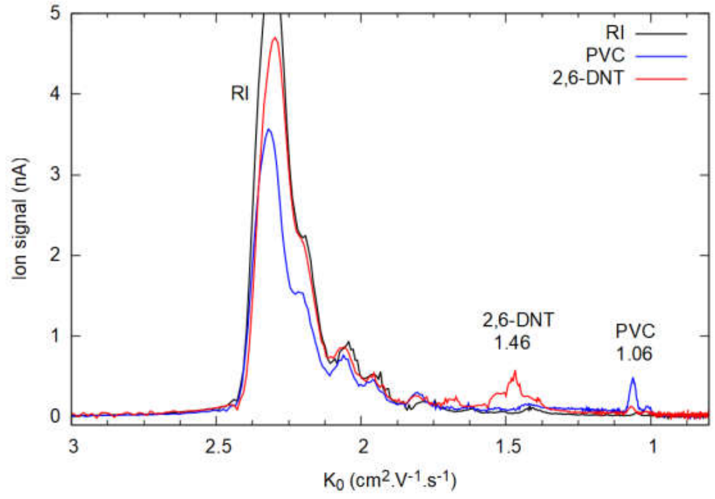

Figure S5.8: The IMS spectrum of 2,6-DNT from PVC.

Section 6. Stainless-steel

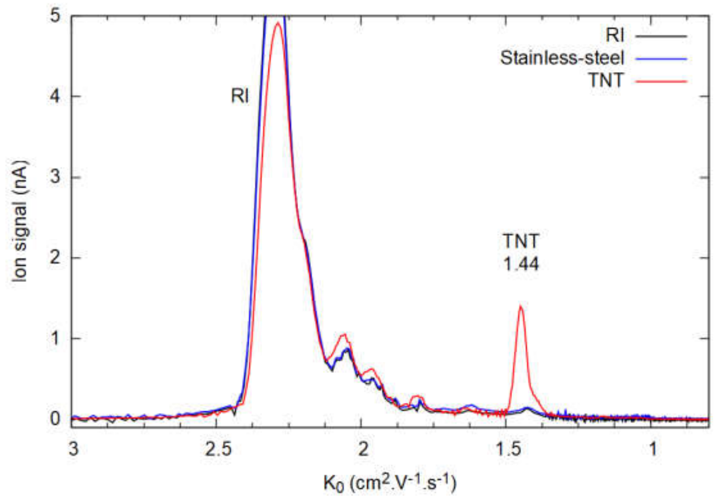

Figure S6.1: The IMS spectrum of TNT from stainless-steel.

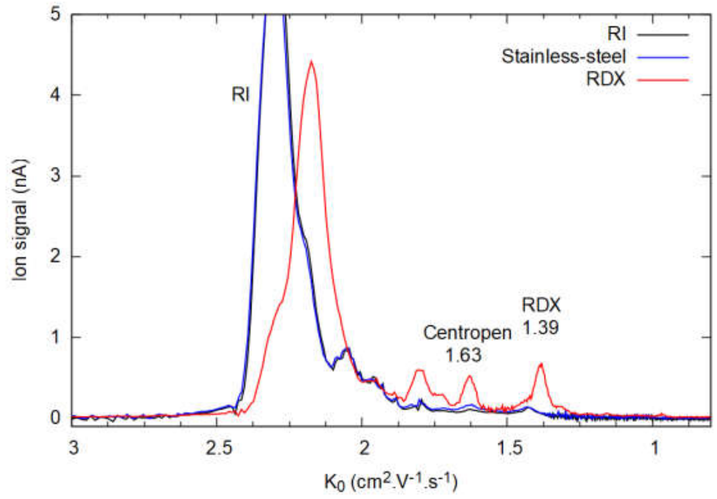

Figure S6.2: The IMS spectrum of RDX from stainless-steel.

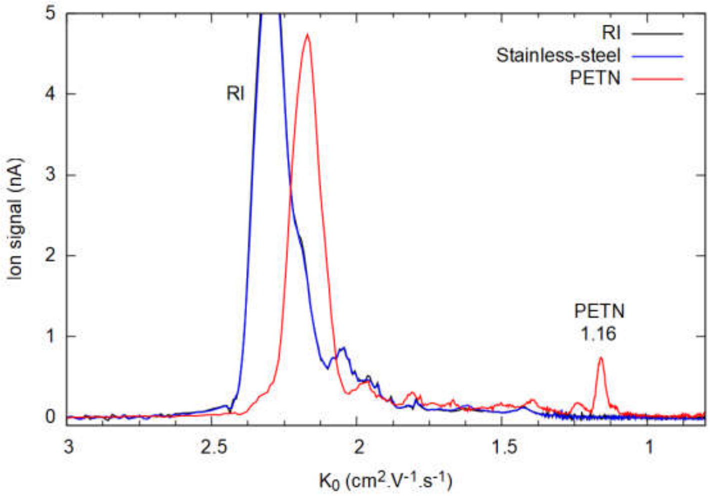

Figure S6.3: The IMS spectrum of PETN from stainless-steel.

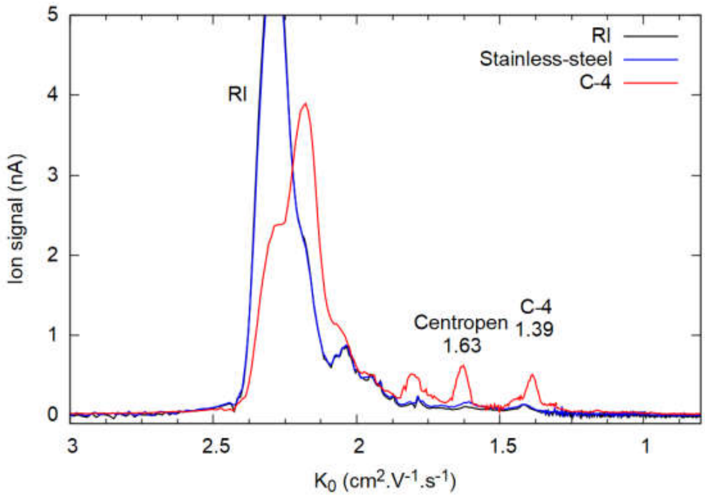

Figure S6.4: The IMS spectrum of C-4 from stainless-steel.

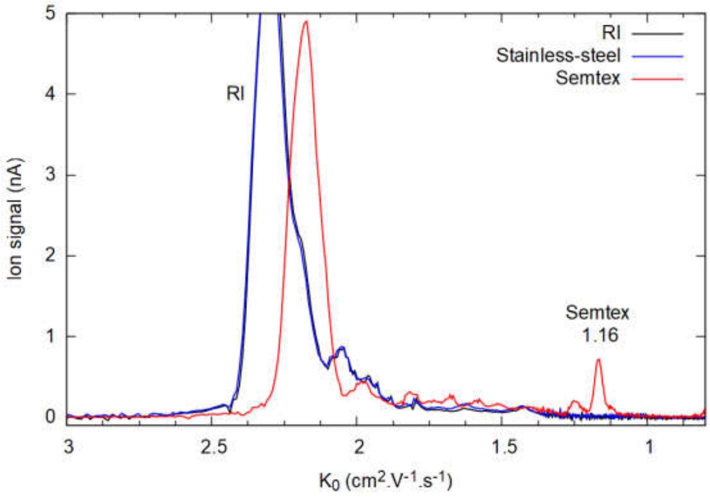

Figure S6.5: The IMS spectrum of Semtex from stainless-steel.

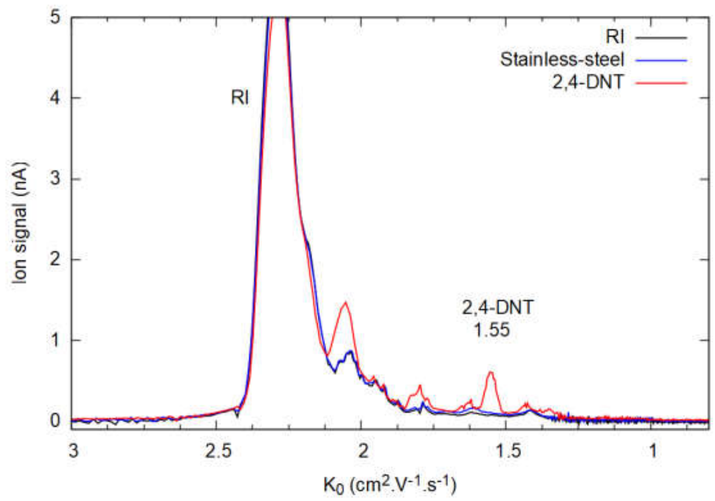

Figure S6.6: The IMS spectrum of 2,4-DNT from stainless-steel.

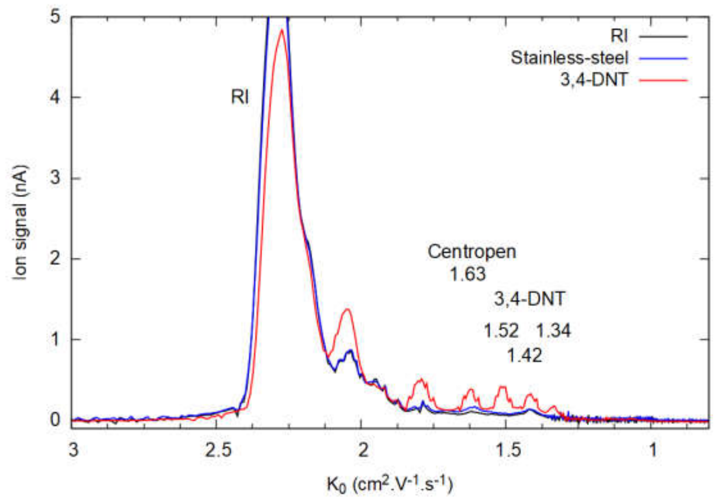

Figure S6.7: The IMS spectrum of 3,4-DNT from stainless-steel.

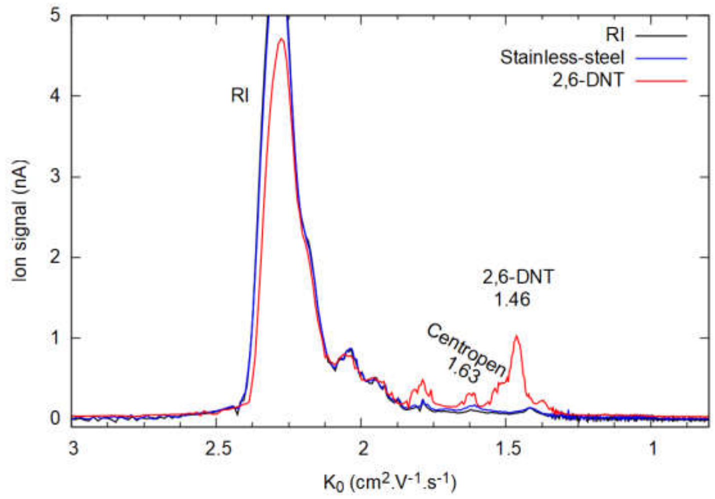

Figure S6.8: The IMS spectrum of 2,6-DNT from stainless-steel.

Section 7. Paper

Possible detection only for PETN and Semtex.

Also, visible a small peak for RDX and C-4.

TNT, 2,4-DNT, 3,4-DNT and 2,6-DNT were not detected.

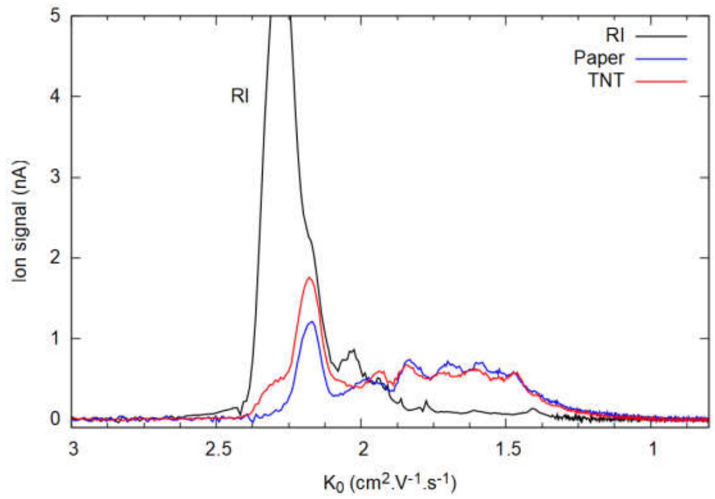

Figure S7.1: The IMS spectrum of TNT from paper.

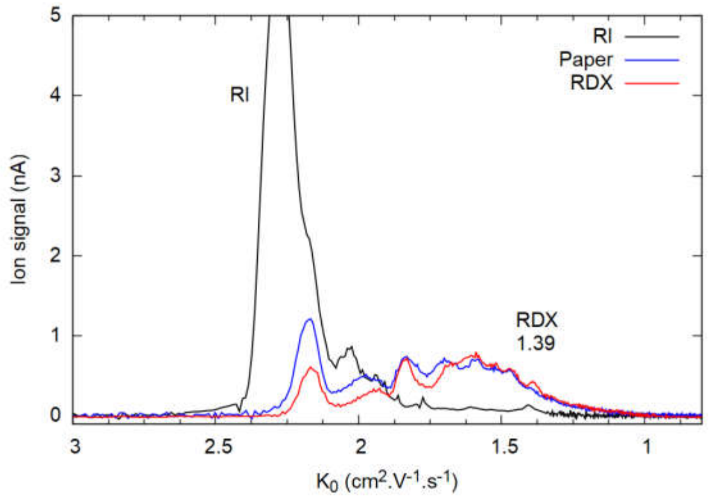

Figure S7.2: The IMS spectrum for RDX form paper.

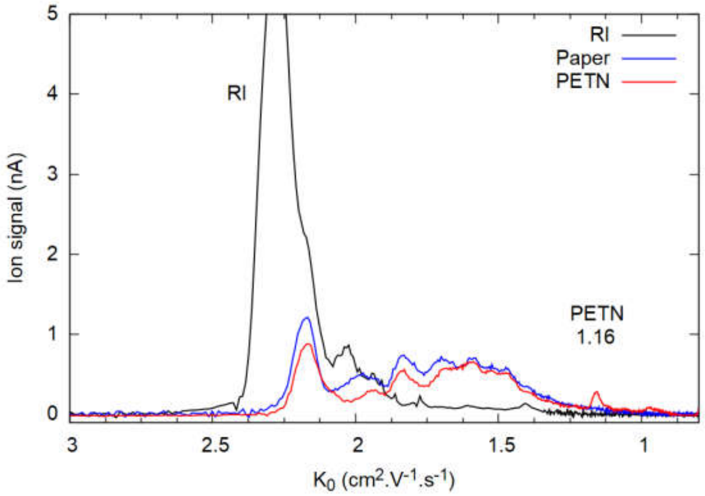

Figure S7.3: The IMS spectrum of PETN form paper.

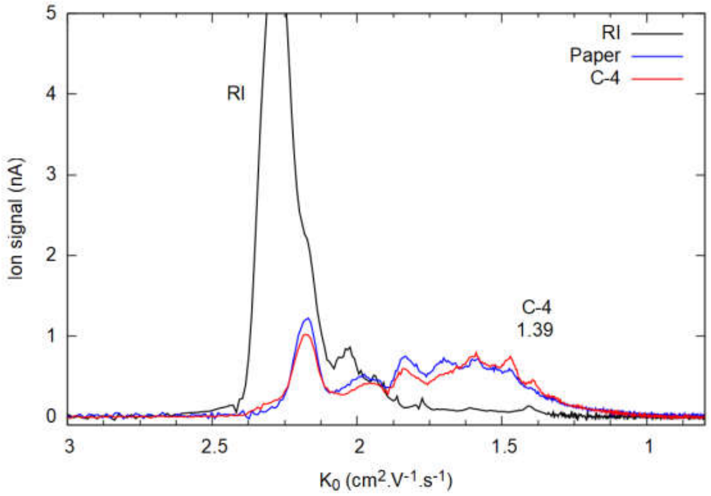

Figure S7.4: The IMS spectrum of C-4 from paper.

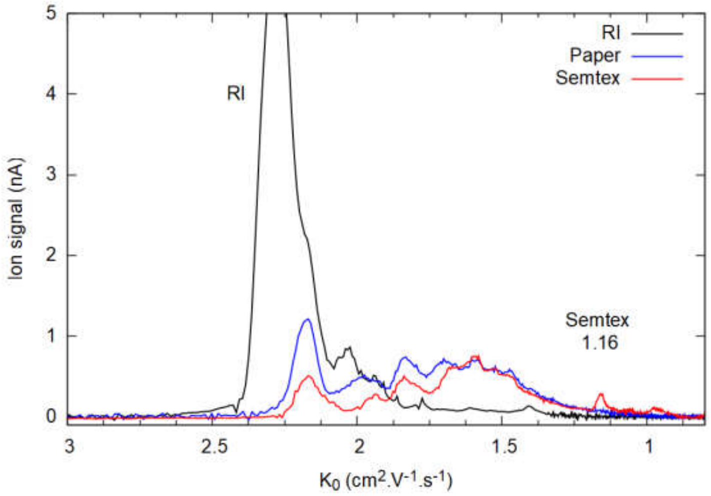

Figure S7.5: The IMS spectrum of Semtex from paper.

Section 8. Wood

No explosives were detected.

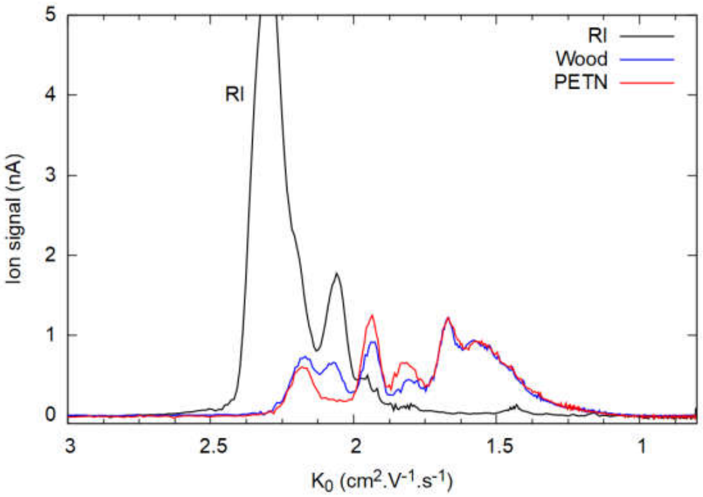

Figure S8.1: The IMS spectrum of PETN from wood.

Section 9.

The IMS spectrum of the marker (Centropen) used for the blackening of the surface area of the examined material.

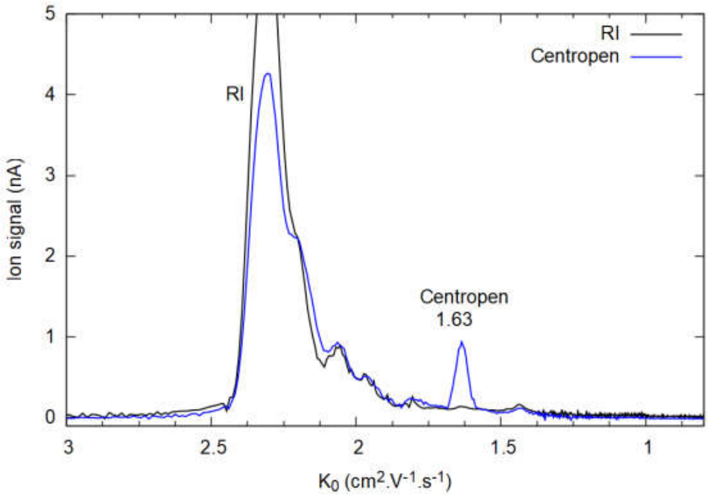

Figure S9.1: The IMS spectrum of marker – Centropen.
